# Andrographolide Exerts Chondroprotective Activity in Equine Cartilage Explant and Suppresses Interleukin-1***β***-Induced MMP-2 Expression in Equine Chondrocyte Culture

**DOI:** 10.1155/2014/464136

**Published:** 2014-10-29

**Authors:** Siriwan Tangyuenyong, Nawarat Viriyakhasem, Siriporn Peansukmanee, Prachya Kongtawelert, Siriwan Ongchai

**Affiliations:** ^1^Department of Companion Animal and Wildlife Clinic, Faculty of Veterinary Medicine, Chiang Mai University, Chiang Mai 50100, Thailand; ^2^Thailand Excellence Centre for Tissue Engineering and Stem Cells, Department of Biochemistry and the Center of Excellence for Innovation in Chemistry, Faculty of Medicine, Chiang Mai University, Chiang Mai 50200, Thailand

## Abstract

Cartilage erosion in degenerative joint diseases leads to lameness in affected horses. It has been reported that andrographolide from *Andrographis paniculata* inhibited cartilage matrix-degrading enzymes. This study aimed to explore whether this compound protects equine cartilage degradation in the explant culture model and to determine its effect on matrix metalloproteinase-2 (MMP-2) expression, a matrix-degrading enzyme, in equine chondrocyte culture. Equine articular cartilage explant culture was induced by 25 ng/mL interleukin-1*β*, a key inducer of cartilage degeneration, in cultures with or without andrographolide ranging from 10 to 50 *μ*M. After 3–21 days, they were analyzed for the markers of cartilage degradation. It was found that interleukin-1*β* increased the release of sulfated glycosaminoglycans and hyaluronan from the explants into the culture media consistently with the decrease in uronic acid and collagen content in the cartilage explants. These catabolic effects were inhibited when cotreated with interleukin-1*β* and andrographolide. In primary equine chondrocytes, andrographolide suppressed interleukin-1*β*-induced MMP-2 mRNA expression and MMP-2 activity in the culture medium. These results confirmed the *in vitro* potent chondroprotective activities of this compound which were performed in cartilage explants and on a cellular level. These may indicate the application of andrographolide for therapeutic use in equine degenerative joint diseases.

## 1. Introduction

Degenerative joint disease (DJD), often called osteoarthritis, is characterized by the progressive and permanent degradation of articular cartilage [1]. Erosion of cartilage can cause joint inflammation and pain, decrease mobility, and reduce performance of athletic humans, dogs, and horses [2]. DJD is the most common cause of lameness and poor performance in equines leading to the reason for culling or early retirement from riding in sports horses [3]. This disease is the result of the metabolic imbalance of cartilage matrix biomolecules which appears in the degradation exceeding synthesis [4]. The catabolic effectors, especially interleukin-1*β* (IL-1*β*), trigger the oversynthesis of matrix metalloproteinases (MMPs), the cartilaginous degrading enzymes, in chondrocytes and the resident synovial cells [5]. These lead to progressive loss of cartilage matrix biomolecules including aggrecan, hyaluronan, and collagen [6]. Inhibition of the mechanism involved in overexpression of these enzymes is the target for development of antiarthritic drugs [7]. Several medicinal plants, especially* Andrographis paniculata,* have been reported to exert potent anti-inflammatory activity and reduce MMPs expression [8–10]. Andrographolide, the major active compound of* A. paniculata*, has been found to protect against inflammatory process leading to downexpression of genes involved in an inflammatory cascade [11]. A recent study in IL-1*β*-treated human chondrocytes found that this compound inhibited the expression of MMP-1, MMP-3, and MMP-13 while increasing the mRNA expression of the natural inhibitors of these enzymes is called tissue inhibitors of matrix metalloproteinase (TIMP) via the NF-*κ*B signaling pathway [12]. These studies led us to suspect that this effectiveness of andrographolide may implicate protective activity against cytokine-induced cartilage degradation in equine. Hence, the present study attempted to investigate the* in vitro* protective effects of andrographolide in equine cartilage explant model and its effects on MMP-2 expression in chondrocyte cultures. The results of this study could provide evidence of the chondroprotective potential of* A. paniculata* for alternative use in horses with DJD.

## 2. Materials and Methods

Andrographolide was purchased from Sigma-Aldrich (Product number 365645). Diacerein was a charitable gift from TRB Chemedica (Switzerland). Reagents for explant and cell culture were from Gibco Cell Culture Media, Invitrogen, USA. IL-1*β* was purchased from R&D Systems, Minneapolis, Minnesota, USA. All other chemicals utilized were of analytical grade.

### 2.1. Cartilage Explant and Chondrocyte Culture Systems

Specimens of articular cartilage were obtained from horses which had died within six to eight hours. Equine articular cartilages were dissected from the weight bearing area of carpal, hock, and fetlock joints of cadaver legs by using aseptic technique. This study was approved for use of animals for scientific purposes and policies by FVM-ACUC on February 1, 2009. Cartilage explants were applied to a method described elsewhere [13]. The explants were incubated in a serum-free Dulbecco's modified Eagle's media (DMEM) containing 200 units/mL of penicillin and 200 *µ*g/mL of streptomycin at 37°C, 5% CO_2_. After 24 hours of incubation, media were collected and stored at −20°C for analysis of the release of cartilage matrix biomolecules such as hyaluronan (HA) and sulfated-glycosaminoglycans (s-GAGs) and designated as the day zero samples. To investigate the effect of andrographolide on the release of HA and s-GAGs into the culture medium, the explants were treated for three days with 25 ng/mL of the recombinant human IL-1*β* to induce cartilage degradation in the absence or presence of andrographolide at the concentrations ranging from 10 to 100 *µ*M. The three-week treatment period was conducted to analyze the remaining UA and collagen in the explant tissues. Treatments were performed in triplicate using tissue from one animal donor. At the end of the incubation, the media and explants were harvested and stored at −20°C for analysis of the matrix degradation.

Equine chondrocytes were isolated by overnight digestion with collagenase (Sigma-Aldrich, type IA) in cartilage at 37°C. The cells were washed with PBS and grown in DMEM containing 10% fetal craft serum as high-density primary monolayer cultures until confluence. The equine chondrocytes at passage three were maintained in serum-free DMEM for 24 hours prior to cotreatment with a range of 10 to 90 *µ*M of andrographolide together with 5 ng/mL recombinant IL-1*β* for 24 hours to investigate the messenger RNA (mRNA) expression of MMP-2. The culture medium was kept at −20°C for gelatinolytic activity assay.

### 2.2. Evaluations for the Markers of Cartilage Matrix Degradation

In the explant model, degradation of the cartilage matrix was investigated using the method clearly reported by Chaiwongsa et al. [14]. The release of s-GAGs and HA in the media was analyzed by dimethylmethylene blue (DMMB) assay and competitive inhibition-based enzyme-linked immunosorbent assay (ELISA), respectively. The explants were digested with papain prior to the determination of UA content by colorimetric assay. Hydroxyproline assay was used for collagen content analysis in the explants. Briefly, a portion of papain-digested samples was further hydrolyzed with 6 N HCl at 90°C for 14 hours then 60°C for 24 hours. Samples (50 *µ*L) were combined with 50 *μ*L of the freshly prepared oxidizing solution for five minutes of incubation at room temperature. They were mixed with 100 *µ*L Ehrlich's reagent prior to incubation for 45 min at 60°C. Samples were extrapolated against hydroxyproline standards at the concentration range of 0 to 10 *µ*g/mL. Absorbance of the mixtures was determined at 540 nm using the Titertek Multiskan M340 multiplate reader.

### 2.3. Assays for MMP-2 Activity

The assay for MMP-2 activity in the conditioned medium by electrophoretic gelatin zymography was conducted as previously described [15]. The samples were subjected to sodium dodecyl sulfate polyacrylamide gel electrophoresis using a 10% acrylamide gel containing 0.1 mg/mL of gelatin under nonreducing conditions. After electrophoresis, the gel was incubated at 37°C in the incubating buffer (50 mM Tris-HCl, 10 mM CaCl_2_, 50 mM NaCl, and 0.05% Brij35, pH 7.6) for 16 hours. Following incubation, the gel was stained with 0.1% Coomassie brilliant blue R250 in 50% (v/v) methanol/10% (v/v) acetic acid and destained with 50% (v/v) methanol/10% (v/v) acetic acid until clear bands over a dark background were observed. The band intensities of gelatinolytic activity were analyzed by using Scion image densitometer and analysis software.

### 2.4. Analysis for MMP-2 mRNA Expression

The level of MMP-2 mRNA from the harvested equine chondrocytes was evaluated by reverse transcription-polymerase chain reaction (RT-PCR) as previously described [16]. Briefly, the total RNA from the harvested equine chondrocytes was extracted by an illustra RNAspin Mini Isolation Kit (GE Healthcare). The evaluation of RNA quality and integrity was conducted as previously described by Jiang et al. [17]. The ratios of A260 nm/A280 nm from the extracted RNA were found in the range of 1.8–2.0, indicating the high purity of the RNA [18]. Prior to the converse of RNA to cDNA, the RNA integrity was evaluated as previously described [19]. The intact 28S and 18S subunits of ribosomal RNA were detected in the agarose denaturing gel with the approximate ratio 2 : 1 (data not shown).

First-strand complementary DNA (cDNA) was synthesized from total RNA using the RevertAid H Minus First Strand cDNA Synthesis Kit (Fermentas). PCR primers for equine MMP-2 were designed based on the nucleotide sequence of* Equus caballus* cDNA (XM_001493281.2). The primers used for RT-PCR were as follows: forward primer: 5′-CAGATCACATACAGGATCATCG-3′, reverse primer: 5′-GTCGGCGTTGCCATACTTCACA-3′ size 321 bp, GAPDH (NM_001163856.1) forward: 5′-TGGTATCGTGGAAGGACTCAT-3′ and reverse: 5′-GTGGGTGTCGCTGTTGAAGTC-3′ size 370 bp. The amplification was performed for 35 cycles and each cycle consisted of denaturing for 45 seconds at 95°C, annealing for 60 seconds at 60°C, and extending for 90 seconds at 70°C. The PCR products were subjected to 1.5% agarose gel electrophoresis. The negative control samples, the absent template sample, and the absent reverse transcriptase sample were performed in parallel. The primer dimer complex was not detectable in the PCR conditions of the present study (data not shown). Quantitative data normalized to GAPDH were obtained using a Scion Image software for PC (Scion Corporation), working in the Gel Plot 2 mode.

### 2.5. Cytotoxicity Assay

The toxicity effects of andrographolide on the cartilage explant system were determined by colorimetric assay, based on the measurement of lactate dehydrogenase (LDH) activity in the culture medium [14]. The culture media of cartilage explants were conducted according to the manufacturer's instruction by comparing the amount of LDH in the samples with the untreated control. The explants treated with 10 mM H_2_O_2_ were used as the positive control.

The cytotoxicity of andrographolide to equine chondrocytes was evaluated by using MTT dye-based cell proliferation and viability assay system according to the instructions of the manufacturer (Calbiochem), as previously described [20]. Yellow (3-(4,5-Dimethylthiazol-2-yl)-2,5-diphenyltetrazolium) bromide) (MTT) was reduced by mitochondrial dehydrogenases in living cells to purple formazan. Briefly, the equine chondrocytes (at a density of 10,000 cells/well) were cultured in the presence or absence of various concentrations of andrographolide for 24 hours. Then, 10 *µ*L of MTT (5 mg/mL) was added, and cells were incubated at 37°C for four hours. At the end of the incubation period, the medium was discarded and cells were washed with PBS; then 100 *µ*L DMSO/well was added to the 96-well plate to solubilize the formazan crystals, followed by utilizing a microplate reader. The percentage of viable cells/well was estimated by the following calculation:

(1) Viability  cells%W=Absorbance  of  treatedAbsorbance  of  control  or  untreated×100.

*Statistical Analysis*. All statistical analyses were performed by using Program R software. Data were expressed as means ± SD of triplicate independent experiments. Statistically significant values were compared by using one-way analysis of variance (ANOVA). Values of *P* < 0.05 were considered significant.

## 3. Results

### 3.1. Effect of Andrographolide on Viability of Equine Chondrocytes in the Systems of Primary Cells Culture and Cartilage Explant Culture

**Figure 1:**
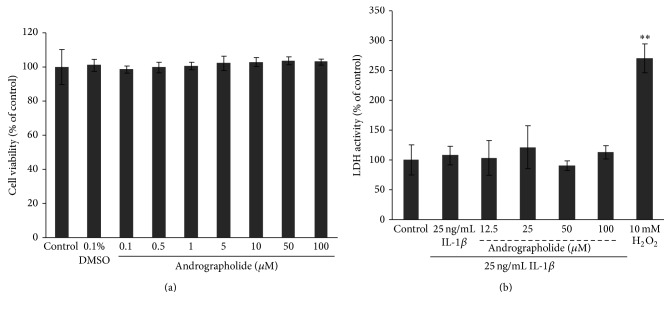
The effect of andrographolide on equine chondrocytes viability. MTT assay and LDH assay were performed in equine primary cell cultures (a) and in equine cartilage explant cultures, respectively (b). The equine chondrocytes were incubated for 24 hours with andrographolide (0.1 to 100 *µ*M), control, and vehicle control (0.1% DMSO in DMEM). The viability of the cells was determined using the MTT assay. The cell viability was expressed as a percentage relative to the control. The bar graphs were expressed as the mean ± SD of three independent experiments. In the equine cartilage explant culture system, the equine cartilage explants were left untreated as the control or treated with IL-1*β* (25 ng/mL) with or without andrographolide (12.5 to 100 *µ*M), respectively. After three days of the treatment period, the culture media were analyzed for LDH release. The results were expressed as a percentage relative to the control. Data were expressed as the mean ± SD of three independent experiments. The significant differences were tested by one-way ANOVA at *P* < 0.001 (∗∗).

Cytotoxicity assays were conducted in order to evaluate whether andrographolide exerts cytotoxicity effect on equine chondrocytes which performed in the conditions of primary cell culture and cartilage explant culture systems. MTT assay, the colorimetric assays based on the reducing potential of the viable cells [20], was used to evaluate the primary culture of the equine chondrocytes.  Figure 1(a) illustrates that the concentration range of andrographolide from 0.1 to 100 *µ*M did not reduce the ability of the cells to metabolize tetrazolium salts. This finding suggested that this concentration range of andrographolide did not interfere with the viability of the primary chondrocytes equine culture. In the cartilage explant culture system, the release of lactate dehydrogenase (LDH) from the damaged cells of the cartilage was investigated using LDH activity assay [21]. As shown in Figure 1(b), neither the explant cultures treated with IL-1*β* nor the cotreatment of IL-1*β* with andrographolide at the concentration up to 100 *µ*M showed any significant changes of the LDH activity compared to the control. The significant release of LDH was demonstrated in the positive control group which was treated by 10 mM H_2_O_2_. The concentrations of andrographolide within this range were therefore selected throughout the study.

### 3.2. Andrographolide Exerted Chondroprotective Activities against IL-1*β*-Induced Cartilage Degradation in Equine Cartilage Explant Culture Model

**Figure 2:**
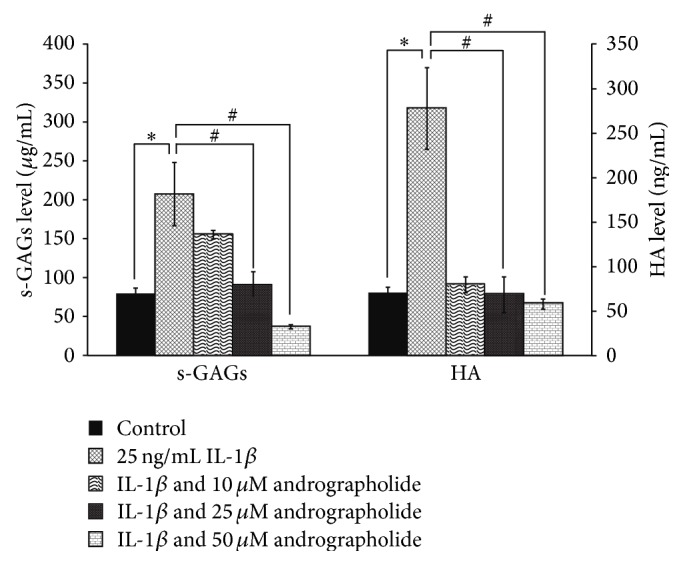
Andrographolide inhibited the release of s-GAGs and HA from equine cartilage explants culture induced by IL-1*β*. The equine cartilage explants were cultured in the conditioned media containing IL-1*β* (25 ng/mL), with or without andrographolide (10 to 50 *µ*M), or left untreated as the control. After three days of the treatment period, the culture medium samples levels of s-GAGs and HA were measured by DMMB assay and competitive inhibition ELISA assay, respectively. Data were expressed as mean ± SD of three independent experiments. The significant differences were tested by one-way ANOVA at *P* < 0.05 (∗, #). Significant different from the control (∗) and IL-1*β* treated group (#), respectively.

IL-1*β*, one of the proinflammatory cytokines, triggers the onset of cartilage degradation by upregulating the expressions of catabolic enzymes, leading to the loss of biomolecules from the cartilage tissue, especially aggrecan, hyaluronan (HA), and collagen [22]. The present study demonstrated the* in vitro* protective effects of andrographolide on IL-1*β*-induced equine cartilage degradation as shown in Figure 2. The equine cartilage explants were cultured in the conditioned media containing IL-1*β*, in the presence or absence of andrographolide. After three days of incubation, the culture media were evaluated for the markers of cartilage degradation such as HA and s-GAGs, a carbohydrate part of aggrecan molecule. The result showed a dramatic increase in the levels of s-GAGs and HA in the culture media of the IL-1*β* treated explants compared to the control. Andrographolide at the doses of 25 and 50 *µ*M significantly suppressed the release of s-GAGs and HA in a dose-dependent manner (Figure 2).

**Figure 3:**
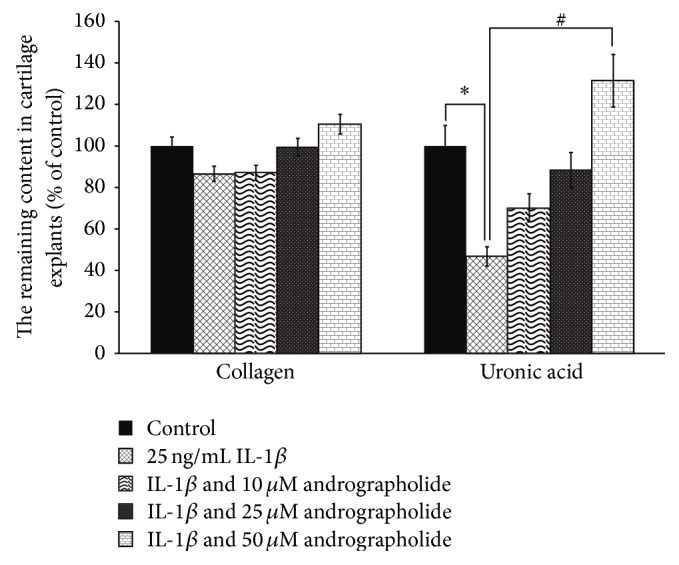
Andrographolide protected the loss of collagen and uronic acid from cartilage explant tissues induced by IL-1*β*. The equine cartilage explants were cultured in the conditioned media containing IL-1*β* (25 ng/mL), with or without andrographolide (10 to 50 *µ*M), or left untreated as the control. After 21 days of treatment period, the cartilage tissues were digested with papain and levels of collagen and UA were measured by colorimetric assays. The results were expressed as a percentage relative to the control. Data are expressed as mean ± SD of three independent experiments. The significant differences were tested by one-way ANOVA at *P* < 0.05 (∗, #). Significant difference from the control (∗) and IL-1*β* treated group (#), respectively.

Parallel to the investigation of cartilage biomolecules which were released into the culture medium, the cartilage explant depletion induced by IL-1*β* was also analyzed by quantitative determination of the remaining collagen and uronic acid (UA), the monosaccharide monomer of s-GAGs of cartilage matrix. The equine cartilage explants were cultured in the conditioned media containing 25 ng/mL IL-1*β* in the presence or absence of different concentrations of andrographolide. After 21 days of incubation, the explant tissues were harvested and digested with papain prior to determination for the remaining of collagen and UA. As shown in Figure 3, the decrease in remaining content of UA and collagen in the explants culture treated with IL-1*β* indicated the enormous loss of these cartilage matrix biomolecules. In the culture wells cotreated with IL-1*β* and andrographolide, it was found that andrographolide was able to counteract the destructive effect of IL-1*β* on the cartilage tissues as indicated by the dose-dependent increase in the content of both UA and collagen (Figure 3).

### 3.3. Andrographolide Suppressed IL-1*β*-Induced MMP2 Expression in Primary Equine Chondrocyte Culture

**Figure 4:**
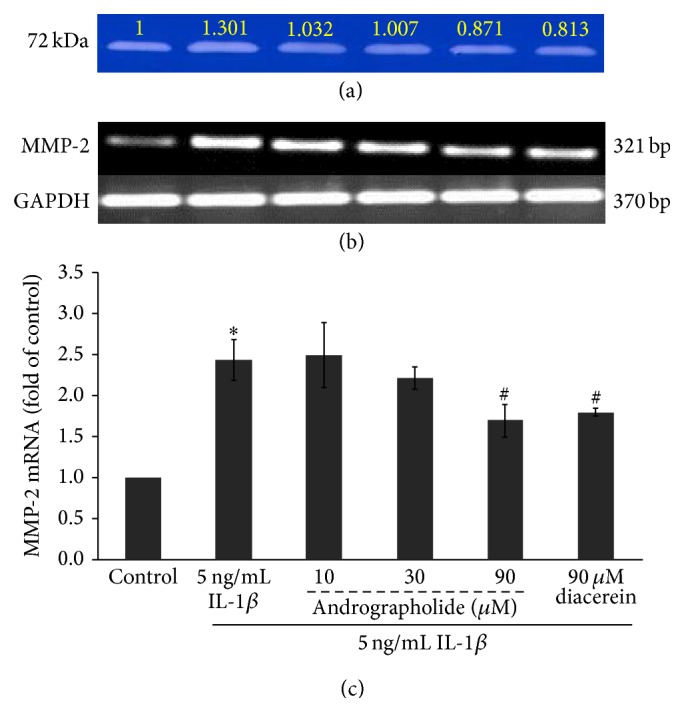
Inhibitory effect of andrographolide on MMP-2 activity and gene expression in equine chondrocyte culture induced by IL-1*β*. The primary equine chondrocytes were cultured in the conditioned media containing IL-1*β* (5 ng/mL), with or without andrographolide (10 to 90 *µ*M), or left untreated as a control. After 24 hours of treatment, the culture medium samples were analyzed for MMP-2 activity by gelatin zymography (a). The numbers above the lanes indicate the fold changes of the enzyme activity compared to the control. The expression of MMP-2 messenger RNA (mRNA) of was performed in the collected cells by semiquantitative RT-PCR; (b) the representative images of agarose gel electrophoresis; (c) the bar graphs indicating relative expression of MMP-2 gene expression as fold change compared to the control. The bar graphs are expressed as mean ± SD of three independent experiments. The significant differences were tested by one-way ANOVA at *P* < 0.05 (∗, #). Significant differences from the control (∗) and IL-1*β* treated group (#), respectively.

On the cellular level of equine chondrocyte, we demonstrated the suppressive effect of andrographolide on expression of MMP-2, one of the catabolic enzymes involved in cartilage degradation, by using semiquantitative RT-PCR technique. The primary equine chondrocytes were incubated with IL-1*β* in the presence or absence of different concentrations of andrographolide for 24 hours. Diacerein, the antiarthritic agent, was parallel treated as the positive control [7]. The increase in of MMP-2 activity in the culture media which was analyzed by gelatin zymography was found to increase in the IL-1*β* treated condition when compared to the control (Figure 4(a)). This effect was decreased when cotreated with andrographolide or diacerein. These results are consistent with the expression of MMP-2 gene (Figures 4(b) and 4(c)). IL-1*β* caused a dramatic increase in MMP-2 mRNA which was significantly suppressed by andrographolide and diacerein at 90 *µ*M.

## 4. Discussion

Articular cartilage damage is the most common phenomena in osteoarthritis and rheumatoid arthritis. The deterioration of cartilage causes pathology and disability in humans and animals. The mechanism of action is involved in the upregulation of proinflammatory cytokines synthesis in chondrocytes and synoviocytes, especially IL-1*β* and TNF-*α* [23, 24]. These catabolic factors interfere in extracellular matrix turnover by accelerating the degradation of cartilage matrix via promotion of the metalloproteinases production such as matrix metalloproteinases (MMPs) and a disintegrin metalloproteinases (ADAMs) [25, 26]. These lead to an increase in degradation and release of matrix degradation products from the cartilage such as hyaluronan, proteoglycans, and collagen [27]. Hyaluronan, also called hyaluronic acid (HA), is a long-chain linear heteropolysaccharide classified as nonsulfated glycosaminoglycan. Aggrecan, the major proteoglycan of cartilage extracellular matrix, consists of about a hundred chains of heteropolysaccharide which mostly are sulfated glycosaminoglycan (s-GAGs) per a core protein. The glycosaminoglycan chains of HA and aggrecan share a similarity of disaccharides repeating units which are composed of uronic acid sugars (UA) and amino sugars [28]. A large numbers of aggrecan monomers aggregated with a long chain hyaluronan, embedded in the collagen network of cartilage matrix, leading to stabilization of the cartilage matrix integrity and its biological role [29]. Therefore, the rapid destruction of the cartilage matrix over the repair synthesis leads to losing the matrix components and tissue functions.

The present study demonstrated the great elevation of hyaluronan and s-GAGs in the culture medium of the IL-1*β* treated group concomitantly with the significant reduction of collagen and UA content in the equine cartilage explants. In cotreatment of IL-1*β* with diacerein, an antiarthritic agent, both these markers of cartilage degradation were decreased (data not shown). Our results indicated that the* in vitro* system of equine cartilage explant degradation induced by IL-1*β* was able to work properly, similar to the previous reports [14, 30]. Based on the results of this study, andrographolide clearly opposed the effects of IL-1*β*-induced equine cartilage explants degradation by reducing the release of s-GAGs and HA, consistent with protecting the loss of collagen and UA. Interestingly, andrographolide at 50 *μ*M was likely to enhance the remaining content of collagen and UA over the control. This may suggest that the compound has effects on synthesis of cartilage biomolecules including collagen, aggrecan, and hyaluronan. Nevertheless, the further investigation is needed to be clearly elucidated before conclusion. In chondrocytes culture model, andrographolide demonstrated the inhibitory effect of IL-1*β*-induced MMP-2 mRNA expression. Although the protein level of MMP-2 was not conducted, activity of this enzyme elevated by IL-1*β* was clearly reduced by andrographolide. Further study the effect of this compound on MMP-2 posttranslational modification which regulated by membrane-type matrix metalloproteinase-1 and tissue inhibitor of metalloproteinase-2 [31] are interested to be explored. Interestingly, the lower doses of andrographolide appeared to significantly suppress cartilage degrading parameters in cartilage explant model compared to those of andrographolide which was required for powerful downregulation of MMP2 expression in primary chondrocyte culture. These suggest that chondrocytes in the intact explant culture and in monolayer culture may exhibit some different response to this compound.

Although MMP-2 is not proposed to play a key role in arthritic cartilage degradation, high level of this enzyme was found in synovial fluid of horses with aseptic joint disease and septic arthritis. It was decreased when the affected horses were successfully treated [32]. It has been reported that the potent stimulators of cartilage degradation such as IL-1*β* and TNF-*α* upregulated MMP-2 gene expression by selectively inducing mitogen-activated protein kinase (MAPK) signaling pathway via MKK3/6-p38, leading to phosphorylation of the activating transcription factor 2 (AP-2) which consequently mediated transcriptional processing of MMP-2 gene [33, 34]. Recently, it has been demonstrated that p38 inhibitors suppressed joint degeneration in animal model of osteoarthritis [35]. In addition, the recent study reported that andrographolide suppressed expressions of MMP-1, MMP-3, and MMP-13 in IL-1*β* stimulated osteoarthritic human chondrocyte culture via NF-kB signal transduction [12]. Consistent with previous studies, these implied that andrographolide has strong chondroprotective activities by suppressing MMPs expression via both main signal transduction pathways of IL-1*β*. Nevertheless, the further study to evaluate the effect of andrographolide on IL-1*β*-induced signals transduction associated with MMP-2 expression in chondrocyte is still required.

Andrographolide, a diterpene lactone, is the major active constituent of* A. paniculata*. It has been shown to have anti-inflammatory activity by suppressing synthesis and activities of proinflammatory cytokine such as IL-1*β*, TNF-*α*, IL-6, IL-10, and COX systems [36–38]. Enhancement of cytokines synthesis consequently provokes genes associated with pathologies in many diseases including rheumatoid arthritis and osteoarthritis [39–41]. It has been reported that the extract of* A. paniculata* which contained 30% andrographolide relieved rheumatoid arthritis symptoms [9]. It was proposed that the compound may suppress the inflammatory process in rheumatoid arthritis via AP-1 and/or STAT3 modulation. Taken all together, we hypothesize that andrographolide may also relieve joint inflammation and exert chondroprotection against cytokines induction via several intracellular signaling including NF-kB and MAPK pathways. The above-mentioned effects of andrographolide result in the decrease of several target genes which are involved in cartilage degradation, especially the matrix metalloproteinases family. The further experimental chondroprotective tests of andrographolide in animal models will be investigated including its effects on activation of cartilage matrix biosynthesis. These will provide more important scientific evidence for supporting the chondroprotective potential of andrographolide.

## 5. Conclusions

The present study demonstrated the potent chondroprotective activities of andrographolide in equine cartilage explants model. These results were supported by the suppressive effects of this compound on IL-1*β*-induced MMP-2 expression in equine chondrocyte cultures. As a result, we may also presume that the involved mechanisms were not only via suppression of MMP-2 expression, but also via other MMPs which are involved in cartilage degradation at the cellular level, leading to diminished degradation of equine cartilage explants which were induced by IL-1*β*. These may suggest the alternative use of* A. paniculata* as antiarthritic agent for degenerative joint diseases in horses and other animals.
